# 

**Published:** 1898-04-09

**Authors:** 


					V
the HOSPITAL. ' "The standard of highest purity."? Lancet. April 9an, 1898.
CADBURY'S ^ fjf w* CADBURY'S
COCOA
ABSOLUTELY PURE. NO DRUGS OR ALKALIES USED.
COCOA
"*a--S3"-,53EgT;; nsrr*~ -"hi Infi'ruiinr
No. 602. Vol. XXIY. [JSg-a Saitodat,
April 9th, 1898.
? ?^ndKTSlKaud Norwich hospi.tal '
[SutecriptionTermB.] paICE TWOPENCE.

				

